# Avian Influenza Risk Perceptions, Laos

**DOI:** 10.3201/eid1307.061197

**Published:** 2007-07

**Authors:** Hubert Barennes, Bertrand Martinez-Aussel, Phengta Vongphrachanh, Michel Strobel

**Affiliations:** * Institut Francophone pour la Médecine Tropicale, Vientiane, Lao PDR; † Ministry of Health, Vientiane, Lao PDR

**Keywords:** Laos, Lao PDR, avian influenza, bird flu, behavior, letter

**To the Editor:** After the 2004 outbreak of highly pathogenic avian influenza (HPAI) in poultry in Lao People’s Democratic Republic (PDR), the Ministry of Health implemented extensive virologic surveillance (*1*,*2*). Surveillance began in July 2005, and by early 2006, only sporadic cases were found. In July 2006, an outbreak of HPAI was confirmed on 2 chicken farms in Vientiane, the capital city of Lao PDR (*1**,**3*). Most of Laos’ ≈20 million chickens are kept on family-owned backyard farms; 3.2 million are on commercial farms (*4*). This production meets 80% of Lao poultry (chicken, duck, goose, quail) needs; imports from neighboring countries, either through legal trade or cross-border smuggling, account for the rest (*3*). Common poultry diseases occur frequently during the cold season, and lack of reporting of poultry deaths is of concern (*4*).

Until February 2007, no human cases of influenza A (H5N1) had been reported in Lao PDR. To learn more about Laotians’ knowledge of HPAI and perceptions of their risk, we conducted a cross-sectional survey.

In March–April 2006, participants in 3 settings (Vientiane, urban; Oudomxay, semiurban; Attapeu Province and Hinheub District, both rural) were interviewed in the Lao language by means of a standardized 33-question survey. We recorded information about behavior, poultry handling and keeping practices, and poultry deaths. We used multivariate analysis (Stata, version 8; Stata Corporation, College Station, TX, USA) to analyze the factors associated with behavior changes.

Using a random sampling list of visitors and vendors, we interviewed 461 respondents in 4 Vientiane city markets (Vientiane has 114,793 households and 3,700 registered poultry farms) (*5*). Semiurban respondents were recruited in Oudomxay (40,987 households, 715 poultry farms), an active trading zone near the Chinese border. Rural respondents were recruited from Hinheup District and in Attapeu (19,050 households, 360 poultry farms), near the Vietnam border. Twenty villages were randomly selected, and 10 participants per village were randomly selected for interview. Approval for the investigation was obtained from the health and market authorities. Oral consent for interview was obtained from participants.

A total of 842 participants were interviewed (Table). Differences in occupation and literacy were associated with different study areas. Differences in participant sex and age were also noted because, in the rural areas, interviews took place in the home. A total of 583 (69.3%) participants were female: 302 (65.5%), 139 (68.2%), and 150 (79.3%), in urban, semiurban, and rural areas, respectively; p = 0.002, 95% confidence interval 66–72. Mean ages for participants in these areas were 41 (range 40–43), 34 (range 32–36), and 38 (range 37–41) years, respectively; p<0.001. Animal breeding was conducted by 50% of families. Daily close exposure to poultry was common (39.6%). Few families owned a henhouse, and no special handling of poultry was reported. Rates of poultry vaccination against common poultry diseases were higher in urban and semiurban areas; veterinary surveillance was low (10.2%).

**Table:** Avian influenza knowledge, risk perception, and poultry-keeping behavior, Lao People’s Democratic Republic*

Characteristic	Urban, n (%)	Semiurban, n (%)	Rural, n (%)	Total, n (%)	p value	95% CI
Total persons interviewed	461	192	189	842		
Illiterate	175 (37.9)	60 (31.2)	181 (95.7)	416 (49.4)	<0.001	47.1–54
Occupation						
Housewife	126 (27.3)	24(12.5)	94 (50)	244 (28.9)	<0.001	32–25.9
Farmer	25 (5.4)	36 (18.75)	75 (40)	136 (16.1)	<0.001	13.7–18.6
Government worker	103 (22.4)	22 (11.5)	3 (1.5)	128 (15.2)	<0.001	12.8–17.6
None	2 (4.3)	0	24 (12.6)	36 (4.2)	<0.001	2.9–5.6
Keep poultry	185 (40.2)	97(50.5)	159 (84.3)	441 (59.4)	<0.001	19 (17–20)
>1 poultry death, past 2 mo†	58 (31.3)	84 (86.5)	95 (59.7)	239 (54.1)	<0.000	49.5–58.8
Any poultry deaths, past 2 y	95 (51.3)	62 (63.9)	141 (88.6)	298 (65.5)	<0.001	63.2–71.9
Response to dead poultry (n = 399)‡						
Bury dead chickens	105 (56.7)	87 (89.6)	118 (74.2)	310 (70.2)	<0.001	66–74.6
Throw out dead chickens	50 (27.0)	5 (5.1)	9 (5.6)	64 (14.5)	<0.001	11.2–17.8
Eat dead chickens	1 (0.5)	2 (2.0)	7 (4.4)	10 (2.2)	0.06	0.9–3.7
Treat other chickens	0	0	5 (2.6)	5 (0.5)	<0.001	0.07–1.1
Apply lime to backyard	0	8 (1.7)	1 (0.5)	9 (1.0)	<0.001	0.03–1.7
Sell dead chickens	0	1 (1.0)	0	1 (0.1)	0.1	0.00–0.3
Report dead chickens	0	0	0	0	NA	NA
Poultry location						
Henhouse	39 (21.0)	4 (4.4)	7 (4.4)	50 (11.3)	<0.001	8.4–14.3
Inside house	8 (4.3)	1 (1.03)	2 (12.6)	11 (2.4)	0.003	1–3.9
Near house (<5 m)	78 (42.2)	59 (61)	28 (17.7)	165 (37.4)	<0.001	32.9–41.9
Far from house (>5 m)	58 (31.3)	30 (31)	114 (71.7)	202 (45.8)	<0.001	41.2–50.5
Regular poultry vaccination	81 (43.7)	54 (55.6)	19 (11.9)	154 (34.2)	<0.001	30.5–39.4
Information source						
Never heard	8 (1.7)	11 (5.1)	7 (3.7)	26/837 (3.1)	0.02	1.9–4.3
Heard from television	388 (86.4)	158 (87.8)	178 (97.8)	724 (89.2)	<0.001	(86.4–90.8)
Heard from radio	19 (4.2)	12 (6.6)	4 (2.2)	35 (4.3)	0.1	(3.02–5.9)
Read in paper	6 (1.3)	1 (0.5)	0	7 (0.8)	0.003	(0.34–1.8)
Perceive risk for avian influenza						
In Laos	369 (81.6)	110 (60.7)	8 (4.3)	487 (59.6)	<0.001	56.3–63
At home	293 (64.8)	72 (40.0)	5 (2.6)	370 (45.7)	<0.001	41.9–48.8
Unable to describe human disease	116 (25.6)	116 (63.7)	182 (97.5)	414 (50.7)	<0.001	47.3–54.2
Able to describe as lethal for poultry	306 (67.5)	90 (49.7)	2(1.0)	398 (48.7)	<0.0001	45.3–52.2
Behavior change‡	416 (91.8)	125 (69.0)	7 (3.8)	548 (67.1)	<0.0001	63.9–70.4
Stopped eating chicken	328 (72.4)	120 (66.2)	0	448 (54.9)	<0.000	51.5–58.3
Avoided contact	348 (76.8)	60 (33.1)	3 (1.6)	411 (50.3)	<0.000	46.9–53.8
Stopped keeping poultry	335 (73.9)	13 (7.1)	1 (0.5)	349 (42.7)	<0.000	39.4–46.2
Wore mask	338 (74.6)	10 (5.5)	1 (0.5)	349 (42.7)	<0.000	39.4–46.2
Washed hands after contact	100 (22.0)	3 (1.6)	1 (0.5)	104 (12.7)	<0.002	10.5–15
Ate well-cooked chicken	155 (34.2)	3 (1.6)	1 (0.5)	159 (19.4)	<0.000	16.8–22.2

*CI, confidence interval; NA, not applicable. †Mean nos. of poultry deaths were 15 (range 10–19), 27 (range 22–32), and 15 (range 13–18) for urban, semiurban, and rural areas, respectively. Total mean = 19.3; p<0.0001; 95% CI, 17.0–18.4. ‡95% CIs were 89–94, 62–76, and 1–7 for urban, semiurban, and rural areas, respectively.

Overall, 96.9% of respondents had already heard of HPAI, mainly through television. Urban residents ranked it as the most well-known poultry disease, but rural residents ranked it fifth. Less than half of the respondents had some knowledge of the disease signs and symptoms for humans and poultry; 28.4% could describe 1 symptom. Half of the respondents believed that they were not at risk for human avian influenza or that their poultry were not at risk for it. Respondents in urban and semiurban areas knew more about avian influenza than those in rural areas.

During the cold season, poultry deaths were higher in the north (colder) and south than in Vientiane. The poultry mortality rate during the cold season was similar to that of Cambodia (*6*). Behavior regarding poultry deaths differed between areas. Despite a high rate of poultry deaths, none of the interviewees had notified authorities. Since hearing about HPAI, 67.1% respondents, mainly in Vientiane, claimed that they had changed behavior regarding poultry. Multivariate analysis showed the following factors to be associated with behavior change: level of education (p = 0.002), urban living (p<0.001), knowledge of avian influenza risk (p<0.001) and disease (p<0.001), owning poultry (p<0.001), and being a government worker (p<0.001).

This study had limitations but provides new insights on Laotians’ knowledge and poultry practices with regard to HPAI. Despite a high level of awareness, populations underestimated the risk, particularly those in rural areas. Most respondents were unaware of appropriate poultry-handling measures to reduce risk (*6*). The claimed changes were higher (more frequent and more substantial) in urban (91.8%) than in rural sites (3.8%, p<0.001), higher than changes made by their counterparts in Thailand (*7*)*,* and confirmed by reports after the 2004 outbreaks (*8*,*9*). These differences between urban and rural areas might be explained not only by participant characteristics but also by a lower extent of the awareness campaign in rural areas.

Failure to report poultry deaths should be addressed and has several possible explanations. Farmers are accustomed to common yearly poultry deaths, which are not reported. In the absence of an official compensation statement, farmers may fear income loss from massive poultry culling.

Our results emphasize the need for more accurate information about transmission risks, notification requirements, safer behavior and practices, and compensation for losses. Focus also needs to be placed on building capacity in the veterinary system (*10*). These issues should be integrated in the Laos National Avian Influenza Control and Pandemic Preparedness Plan (2006–2010).

## References

[1] Boltz DA, Douangngneun B, Sinthasack S, Phommachanh P, Rolston S, Chen H, H5N1 influenza viruses in Lao People’s Democratic Republic. Emerg Infect Dis. 2006;10:1593–4. 10.3201/eid1210.060658 PMC3290961 17176581

[2] Witt CJ, Malone JL. A veterinarian’s experience of the spring 2004 avian influenza outbreak in Laos. Lancet Infect Dis. 2005;5:143–5. 15766647 10.1016/S1473-3099(05)01305-8

[3] World Health Organization. Avian influenza update no. 56: 15 August 2006. [cited 2007 Apr 18]. Available from http://www.wpro.who.int/NR/rdonlyres/13C8C409-2438-4426-A5E3-7339564D1731/0/AIWeekly56WPRO.pdf

[4] Food and Agriculture Organization. Epidemiology of H5N1 influenza in Asia and implications for regional control. [cited 2007 Apr 18]. Available from http://www.fao.org/ag/againfo/subjects/documents/ai/HPAI-Masseyreport.pdf

[5] National Statistical Centre. Lao statistical yearbook 2003. Vientiane (Lao PDR): Committee for Planning and Cooperation; 2004.

[6] Vong S, Coghlan B, Mardy S, Holl D, Seng H, Ly S, Low frequency of poultry-to-human H5N1 virus transmission, southern Cambodia, 2005. Emerg Infect Dis. 2006;10:1542–8. 10.3201/eid1210.060424 PMC3290951 17176569

[7] Takeuchi MT. Avian influenza risk communication, Thailand. Emerg Infect Dis. 2006;12:1172–3. 16848047 10.3201/eid1207.060277 PMC3291070

[8] Food and Agriculture Organization. Livestock report 2006. Rome, 2006. [cited 2007 Apr 18]. Available from http://www.fao.org/docrep/009/a0255e/a0255e00.htm

[9] US Department of Agriculture. Laos poultry and products: avian influenza 2005. Report no. LA5001. [cited 2007 Apr 18]. Available from http://www.fas.usda.gov/gainfiles/200503/146119131.doc

[10] World Organization for Animal Health. Laos 2002, veterinarians and technical personnel. [cited 2007 Apr 18]. Available from http://www.oie.int/hs2/gi_veto_pays.asp?c_pays=106&annee=2002

